# Targeting arginase-II protects mice from high-fat-diet-induced hepatic steatosis through suppression of macrophage inflammation

**DOI:** 10.1038/srep20405

**Published:** 2016-02-05

**Authors:** Chang Liu, Angana G. Rajapakse, Erwin Riedo, Benoit Fellay, Marie-Claire Bernhard, Jean-Pierre Montani, Zhihong Yang, Xiu-Fen Ming

**Affiliations:** 1 Vascular Biology, Department of Medicine, Division of Physiology, University of Fribourg, Chemin du Musée 5, CH-1700 Fribourg, Switzerland; 2 Laboratory HFR, Hospital Fribourgeois, Chemin des Pensionnats 2, 1708 Fribourg, Switzerland; 3 Promed Medical Laboratory SA, Fribourg, Switzerland; 4 National Center of Competence in Research “Kidney.CH”, Switzerland

## Abstract

Nonalcoholic fatty liver disease (NAFLD) associates with obesity and type 2 diabetes. Hypoactive AMP-activated protein kinase (AMPK), hyperactive *m*ammalian *t*arget *o*f *r*apamycin (mTOR) signaling, and macrophage-mediated inflammation are mechanistically linked to NAFLD. Studies investigating roles of arginase particularly the extrahepatic isoform arginase-II (Arg-II) in obesity-associated NAFLD showed contradictory results. Here we demonstrate that Arg-II^−/−^ mice reveal decreased hepatic steatosis, macrophage infiltration, TNF-α and IL-6 as compared to the wild type (WT) littermates fed high fat diet (HFD). A higher AMPK activation (no difference in mTOR signaling), lower levels of lipogenic transcription factor SREBP-1c and activity/expression of lipogenic enzymes were observed in the Arg-II^−/−^ mice liver. Moreover, release of TNF-α and IL-6 from bone marrow-derived macrophages (BMM) of Arg-II^−/−^ mice is decreased as compared to WT-BMM. Conditioned medium from Arg-II^−/−^-BMM exhibits weaker activity to facilitate triglyceride synthesis paralleled with lower expression of SREBP-1c and SCD-1 and higher AMPK activation in hepatocytes as compared to that from WT-BMM. These effects of BMM conditioned medium can be neutralized by neutralizing antibodies against TNF-α and IL-6. Thus, Arg-II-expressing macrophages facilitate diet-induced NAFLD through TNF-α and IL-6 in obesity.

Nonalcoholic fatty liver disease (NAFLD) referred to as an intracellular accumulation of triglycerides in the liver (steatosis) with or without hepatitis can lead to liver fibrosis and cirrhosis and may contribute to development and progression of the most important chronic complications of diabetes, including cardiovascular disease and chronic kidney disease^1^,^2^. NAFLD is commonly associated with obesity, insulin resistance and type 2 diabetes^3^. Under the metabolic disorder conditions, hepatic *de novo* lipogenesis is paradoxically augmented despite insulin resistance^4^,^5^, which involves various mechanisms including activation of *m*ammalian *t*arget of *r*apamycin *c*omplex 1 - small ribosomal protein S6 kinase 1 (mTORC1-S6K1) signaling and inhibition of 5′ adenosine monophosphate-activated protein kinase (AMPK) pathway, leading to up-regulation and activation of hepatic sterol regulatory element-binding protein-1c (SREBP-1c)^4^,^5^,^6^,^7^. SREBP-1c is a master transcription factor regulating expression of various enzymes in the lipogenesis pathway such as malic enzyme (ME), glucose-6-phosphate dehydrogenase (G6PDH), acetyl-CoA carboxylase-1 (ACC1), fatty acid synthase (FAS), and stearoyl-CoA desaturase-1 (SCD-1)^8^. While mTORC1 pathway has been shown to induce SREBP-1c and promote hepatic lipogenesis^5^,^9^, activation of AMPK pathway e.g., by the antidiabetic drug metformin and thiazolidinediones, accounts for its therapeutic effects on liver steatosis through inhibition of SREBP-1c^7^,^10^,^11^.

It is well recognized that obesity and inflammation are highly integrated processes in the pathogenesis of insulin resistance, type 2 diabetes and NAFLD^12^. Macrophages are heterogeneous and two extreme phenotypes, i.e., pro-inflammatory M1 and anti-inflammatory M2, are the most well investigated^13^. Macrophage infiltration accompanied by inflammation and metabolic alteration occurs in both adipose tissues and liver, contributing to insulin resistance, type 2 diabetes and NAFLD^14^,^15^,^16^,^17^. Recent studies provide convincing evidence that resident macrophages in the liver, i.e., Kupffer cells, are activated towards M1 phenotype in obesity^18^. Moreover, pro-inflammatory macrophages are also recruited to the liver under high fat diet (HFD) feeding^19^,^20^. These studies demonstrate that both activated M1 Kupffer cells and recruited macrophages in the liver cause hepatic inflammation and promote hepatic steatosis.

The regulatory mechanisms of macrophage phenotypes are complex and far from being characterized. Among other markers, iNOS and type-I arginase (Arg-I), the enzymes that are involved in L-arginine/NO metabolism, are associated with M1 and M2 macrophage phenotype, respectively^13^. A type-II arginase (Arg-II) also exists. In contrast to Arg-I which is constitutively and most abundantly expressed in hepatocytes^21^,^22^ and functions as a detoxifying enzyme in urea cycle to remove ammonia^23^, Arg-II is not expressed in hepatocytes but in many extrahepatic tissues/cells including kidney, brain, vasculature, and macrophages^22^,^24^. Moreover, Arg-II is up-regulated in macrophages by pro-inflammatory stimuli such as lipopolysaccharide (LPS) and associated with M1 phenotype^25^. Our recent study provides evidence demonstrating that Arg-II promotes macrophage pro-inflammatory responses, contributing to atherogenesis and obesity-associated insulin resistance^26^. Furthermore, we also showed that Arg-II forms a positive regulatory circuit with mTORC1-S6K1 and negatively regulates AMPK, resulting in functional changes of vascular cells, including eNOS-uncoupling, cell apoptosis/senescence, and impaired autophagy function^27^,^28^,^29^.

Despite the beneficial effects of Arg-II deficiency on the chronic inflammatory diseases, it is to note that pharmacological inhibition of arginase or genetic ablation of Arg-II in HFD-induced obesity mouse model yielded completely contradictory results on the pathology of liver steatosis as reported by the most recently published two studies^30^,^31^. Hence, it is important to further delineate the role of Arg-II in obesity-associated NAFLD and the underlying mechanisms. In this study, we demonstrate that Arg-II deficiency protects against obesity-associated NAFLD in mice through suppression of liver macrophage-mediated pro-inflammatory responses resulting in improved AMPK activation and decreased lipogenesis in hepatocytes.

## Results

### Reduced liver weight in obese Arg-II^−/−^ mice fed HFD

**Figure 1:**
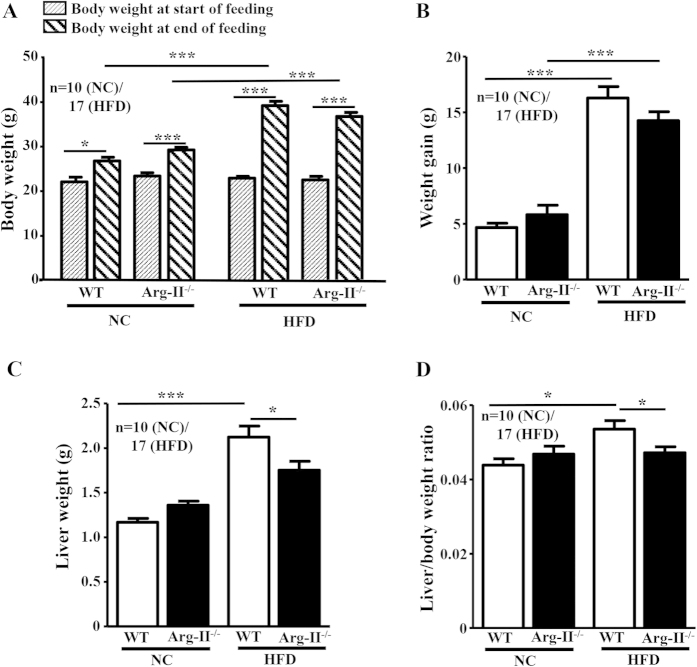
Liver weight is reduced in obese Arg-II^−/−^ mice. Starting from 7 weeks of age, male wild-type (WT) and Arg-II^−/−^ mice were fed a HFD for 14 weeks to induce obesity or maintained on normal chow diet (NC) as controls. **(A)** Body weight was measured at the start and at the end of HFD or NC feeding. **(B)** Weight gains during HFD feeding period. **(C)** Liver weight at the end of HFD. **(D)** Ratio of the liver weight and body weight at the end of HFD. *p < 0.05 and ***p < 0.001 between the indicated groups.

After HFD feeding for 14 weeks, no significant difference in body weight and body weight gain between the WT and Arg-II^−⁄−^ mice were observed (Fig. 1A,B). The liver weight and the liver weight/body weight ratio were, however, significantly lower in the Arg-II^−⁄−^ mice as compared to the WT mice on HFD feeding (Fig. 1C,D).

### Arg-II deficiency reduces HFD–induced liver steatosis

**Figure 2:**
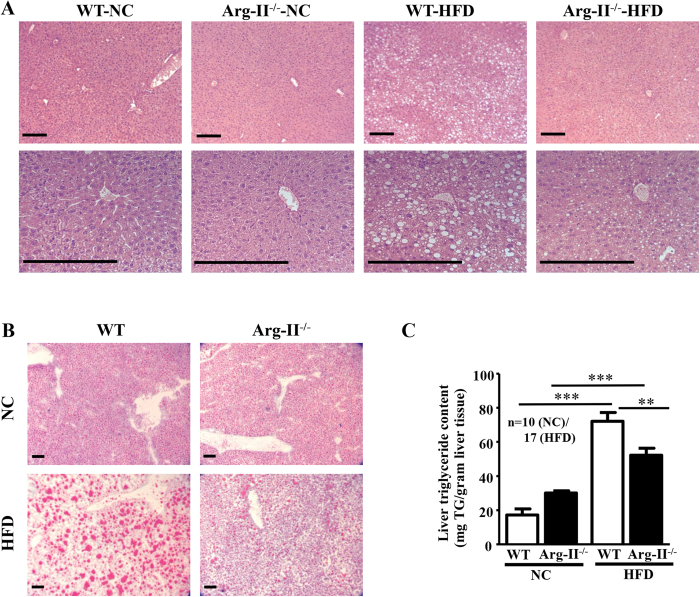
HFD-induced hepatic steatosis is reduced in Arg-II^−/−^ mice. Presented are images of **(A)** HE staining taken at two different magnifications. **(B)** Oil Red O staining in liver of WT and Arg-II^−/−^ mice on either normal chow diet (NC) or high fat diet (HFD). Scale bar = 0.2 mm. **(C)** Liver triglyceride content. **p < 0.01, ***p < 0.001 between the indicated groups.

In line with above observations, Arg-II^−/−^ mice on HFD feeding displayed reduced hepatic steatosis as revealed by histomorphometric analysis of hematoxylin and eosin (HE) staining (Fig. 2A) and Oil Red O staining (Fig. 2B) in the liver sections, and significantly reduced hepatic triglyceride content when compared to the WT mice on HFD (Fig. 2C).

### Arg-II deficiency suppresses hepatic lipogenic enzymes and signaling

**Figure 3:**
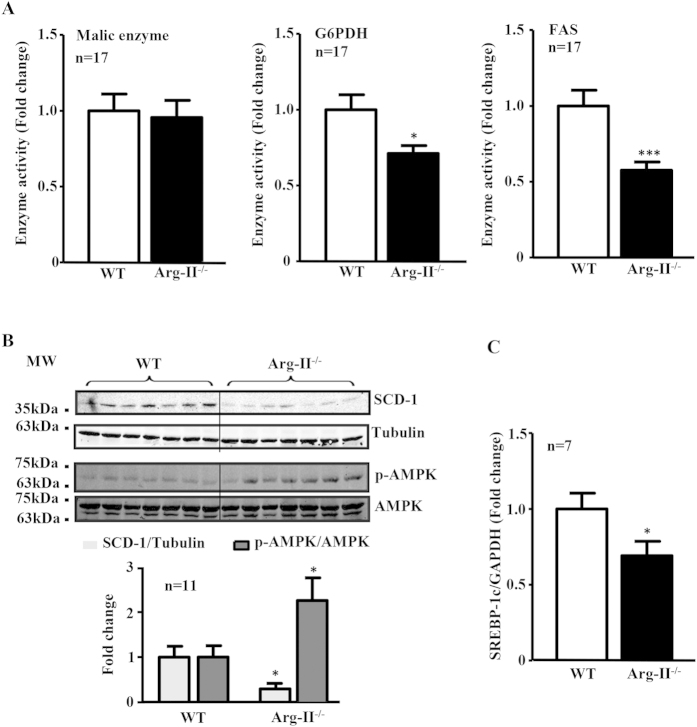
Reduced lipogenic enzyme activity/expression and SREBP-1c expression, and enhanced AMPK signaling in obese Arg-II^−/−^ mice. **(A)** Activity of malic enzyme (ME), glucose-6-phosphate-dehydrogenase (G6PDH) and FAS in liver from obese WT and Arg-II^−/−^ mice. **(B)** Shown is immunoblotting analysis of SCD-1 and phosphorylated AMPK-T172 (p-AMPK) and total AMPK levels from obese WT and Arg-II^−/−^ liver. Tubulin served as a loading control. Quantification of the signals is presented in the graphics below. **(C)** qRT-PCR analysis of SREBP-1c mRNA expression in liver from obese WT and Arg-II^−/−^ mice. *p  < 0.05 and ***p  < 0.001 vs WT-HFD.

Next, we examined the hepatic lipogenic signaling and enzymes. Arg-II^−/−^ mice on HFD feeding revealed decreased activities of lipogenic enzymes in the liver such as G6PDH and FAS but not ME (Fig. 3A). Also the hepatic SCD-1 levels were significantly lower in the Arg-II^−/−^ mice as compared to the WT littermates on HFD feeding (Fig. 3B). The reduced SCD-1 levels in the Arg-II^−/−^ mice were associated with increased AMPK activation as measured by enhanced p-AMPK levels (Fig. 3B). Furthermore, expression of SREBP-1c was significantly reduced in the liver of Arg-II^−/−^ mice on HFD (Fig. 3C). No difference in S6K1 activity as monitored by S6-S235/236 level was observed between the WT and Arg-II^−/−^ mice fed HFD (data not shown). The results demonstrate a role of Arg-II in hepatic lipogenesis linking to the development of liver steatosis induced by HFD feeding. Taking into account that inhibition of SCD-1 in the context of the overflow of free fatty acids coming to the liver on HFD feeding has been shown to cause the buildup of toxic lipids resulting in increased hepatocyte cell death and liver injury despite the decreased TG deposition^32^,^33^, we assessed the liver injury by monitoring plasma alanine aminotransferase (ALT) and aspartate aminotransferase (AST). As shown in Fig. S1, plasma ALT and AST are significantly increased upon HFD feeding in both WT and Arg-II^−/−^ mice. However, the increase in plasma ALT and AST tends to be smaller in obese Arg-II^−/−^ mice as compared to obese WT mice.

### Arg-II-deficiency enhances adiponectin levels in adipose tissue, but not in plasma and in liver

**Figure 4:**
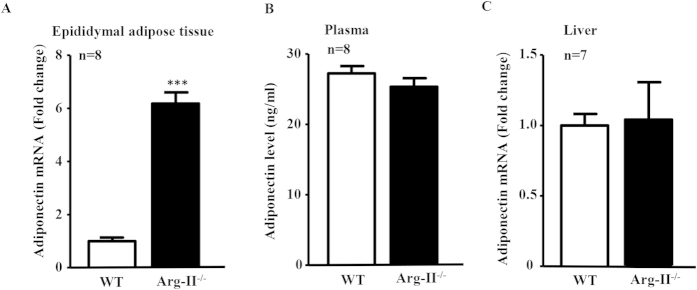
Enhanced adiponectin in fat tissue but not in plasma or liver in Arg-II^−/−^ mice. **(A)** qRT-PCR analysis of adiponectin mRNA expression in epididymal fat tissue from obese WT and Arg-II^−/−^ mice. ***p < 0.001 vs WT-HFD group. **(B)** Adiponectin level in plasma measured by ELISA. **(C)** qRT-PCR analysis of mRNA expression of adiponectin in liver tissue from obese WT and Arg-II^−/−^ mice.

Taking into account that Arg-II is an extrahepatic isoform of arginase and was not detectable in WT mouse liver and that Arg-I expression was not affected by Arg-II-deficiency (Fig. S2), the protective effect of Arg-II-deficiency on HFD-induced liver steatosis must be secondary to its effect on Arg-II-expressing cells/tissue. Given that adiponectin exerts its protective effects against liver steatosis through activating AMPK signaling pathway^34^ which is enhanced in Arg-II^−/−^ mouse liver (Fig. 3B), we examined the adiponectin levels in adipose tissues, plasma and liver. Though the adiponectin mRNA level in epididymal adipose tissue as monitored by qRT-PCR is indeed significantly increased in Arg-II^−/−^ mice (Fig. 4A), no difference in adiponectin levels was found in plasma or liver between WT and Arg-II^−/−^ mice on HFD (Fig. 4B,C). No difference in weight of epididymal adipose tissue was found between WT and Arg-II^−/−^ mice on HFD either (Fig. S3). These results rule out the endocrine and paracrine effects of adiponectin as a mechanism for enhanced AMPK and reduced hepatic lipogenesis in the Arg-II^−/−^ mice.

### Arg-II-positive macrophages are increased in fatty liver

**Figure 5:**
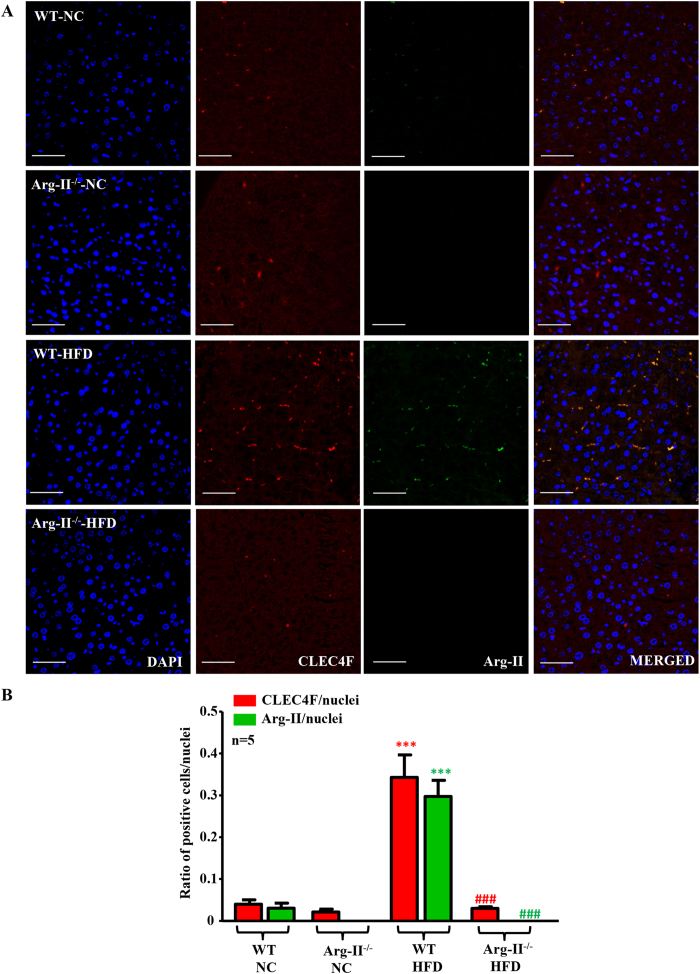
Enhanced hepatic inflammation upon HFD in WT is associated with increased Arg-II expression in macrophages in liver, which is abrogated by Arg-II-deficiency. (**A**) Shown are representative images of immunostaining of the liver sections with macrophage marker CLEC4F (red) and Arg-II (green) followed by nuclei counterstaining with DAPI (blue). Scale bar = 50 μm. (**B**) Quantification of the signal is expressed as a ratio of CLEC4F- or Arg-II-positive cells to tissue nuclei number. ***P < 0.001 vs WT-NC. ^###^P < 0.001 vs WT-HFD.

**Figure 6:**
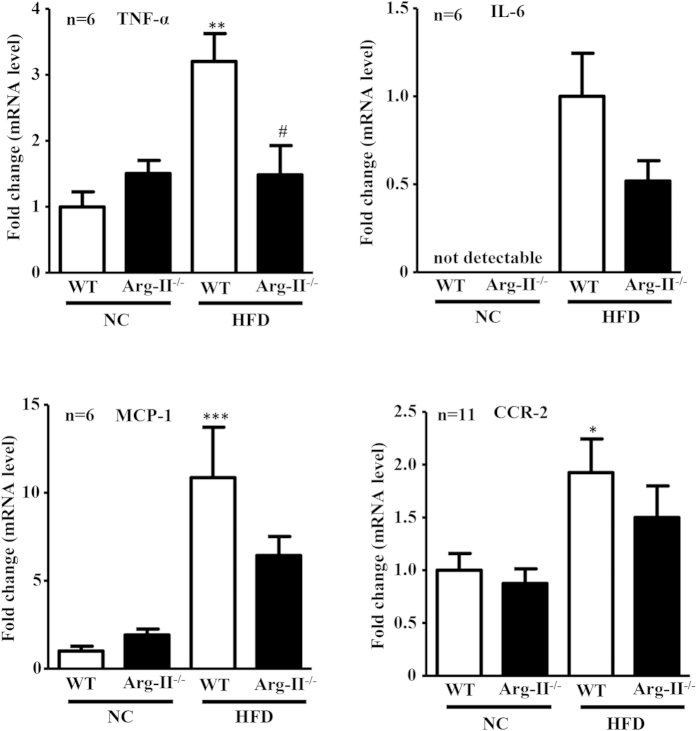
Decreased cytokine production in liver of Arg-II^−/−^ mice. qRT-PCR analysis of mRNA expression of cytokines in liver tissue from WT and Arg-II^−/−^ mice either on NC or HFD. Except for IL-6, all data are presented as fold change from WT-NC group, since IL-6 is not detectable in WT-NC; the data for IL-6 are presented as fold change from WT-HFD group. *p < 0.05, **p < 0.01, ***p < 0.001 vs WT-NC group; #p < 0.05 vs WT-HFD group.

Macrophage-mediated inflammation has been mechanistically linked to the augmented hepatic lipogenesis in obesity^17^. Our previous studies demonstrate that Arg-II-deficiency suppresses obesity-associated systemic macrophage-mediated inflammation^26^. We thus examined the hepatic macrophage inflammation. Under HFD feeding, the number of CLEC4F positive and F4/80 positive macrophages in the WT-liver was increased, which was prevented in Arg-II^−/−^ mice (Fig. 5 and Fig. S4). Moreover, Arg-II was exclusively expressed in these cells and was enhanced on HFD feeding as demonstrated by immunofluorescence staining (Fig. 5). In line with these observations, the pro-inflammatory cytokine TNF-α was significantly lower in obese Arg-II^−/−^ mice liver (Fig. 6). We also observed a lower IL-6, MCP-1 and CCR-2 levels in Arg-II^−/−^ mice liver, which statistically did not reach significance (Fig. 6). These results indicate that suppression of liver steatosis in obese Arg-II^−/−^ mice might be attributable to reduced hepatic macrophage-mediated inflammation.

### Suppressed production of IL-6 and TNF-α in Arg-II^−/−^ macrophages accounts for reduced hepatic lipogenesis

**Figure 7:**
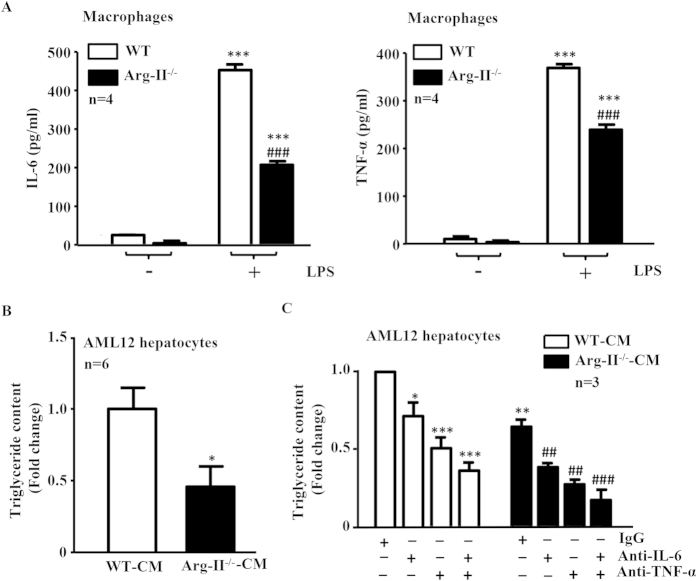
Suppressed production of IL-6 and TNF-α in Arg-II^−/−^ macrophages accounts for reduced lipogenesis in hepatocyte AML12 cells. The serum-starved (overnight) WT- or Arg-II^−/−^-BMM were incubated in the absence or presence of 100 ng/ml LPS for 8 hours, the conditioned medium (CM) was then collected. **(A)** IL-6 and TNF-α production in CM collected from WT- or Arg-II^−/−^-BMMs in the absence or presence of LPS was evaluated by ELISA. ***p < 0.001 vs controls (-LPS), ^###^p < 0.001 vs WT+LPS. **(B)** AML12 hepatocytes were serum-starved for 6 hours in 0.2% bovine serum albumin (BSA)-ITS-DMEM-F-12 Ham medium and then incubated with CM of LPS-treated BMM from WT or Arg-II^−/−^ mice as indicated in the presence of oleic acid (OA, 20 mmol/L) for 24 hours. The lipid was then extracted for measurement of the triglyceride content in the cells. *p < 0.05 vs WT-CM. **(C)** Experiment procedure is the same as in panel B, except that BMM-CM was pre-treated for 2 hours with control rat IgG (10 μg/ml) or neutralizing antibodies anti-IL-6 (18 μg/ml) or anti-TNF-α (0.4 μg/ml) or anti-IL-6 (18 μg/ml) plus anti-TNF-α prior to the addition to the cells. *p < 0.05, **p < 0.01, ***p < 0.001 vs (WT-BMM-CM + IgG); ^##^p < 0.01, ^###^p < 0.001 vs (Arg-II^−/−^ **-**BMM-CM + IgG).

To further confirm the above-mentioned findings, we prepared macrophages derived from bone marrow cells (BMM) that were isolated from WT or Arg-II^−/−^ mice. The levels of secreted TNF-α and IL-6 in the BMM-conditioned medium (CM) from BMM upon LPS stimulation (100 ng/ml, 8 h) were determined. As shown in Fig. 7A, production of TNF-α and IL-6 upon LPS stimulation was significantly reduced in BMM from Arg-II^−/−^ mice as compared to those from WT control animals. We then evaluated the effects of the CM from LPS-treated BMM on triglyceride synthesis in a murine hepatocyte cell line (AML12) in the presence of oleic acid (OA) that enhances hepatic triglyceride synthesis^35^. Remarkably, triglyceride synthesis in the hepatocytes was significantly lower in the presence of Arg-II^−/−^-BMM-CM as compared to WT-BMM-CM (Fig. 7B), suggesting that the lower IL-6 and TNF-α levels released from Arg-II^−/−^ BMM accounts for the reduced triglyceride synthesis induced by Arg-II^−/−^-BMM-CM.

To further reinforce the above conclusion, BMM-CM was first incubated with control IgG or neutralizing antibodies against IL-6, TNF-α or both and then used to treat the AML12 cells in the presence of OA. Fig. 7C shows that anti-TNF-α or anti-IL-6 antibody alone reduced triglyceride synthesis stimulated by BMM-CM of WT or Arg-II^−/−^. The strongest suppressing effects were achieved with the combination of both antibodies, supporting our conclusion drawn above.

### Arg-II^−/−^-BMM-CM elicits enhanced AMPK activation and reduced expression of SREBP-1c and SCD-1 in hepatocytes

**Figure 8:**
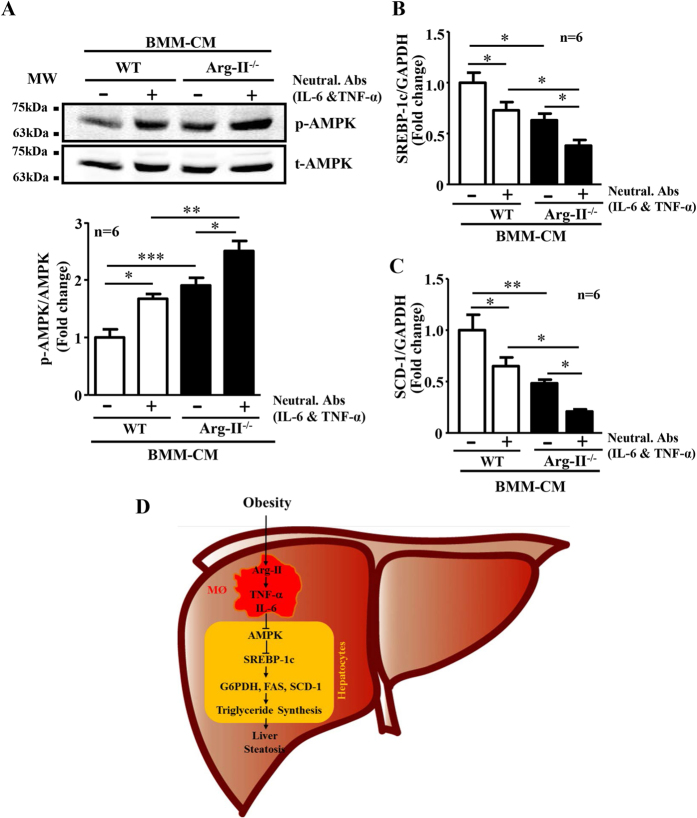
CM from Arg-II^−/−^-BMM elicits increased AMPK signaling and decreased SREPBP-1c and SCD-1 in hepatocytes. Experiment procedure is the same as in Fig. 7B, except that **(A)** AML12 hepatocyte cell lysates were prepared for immunoblotting analysis of AMPK activation. Quantification of the signals is presented below. **(B,C**) RNA was extracted for qRT-PCR analysis of mRNA expression of SREBP-1c and SCD-1. For all the panels *p < 0.05, **p < 0.01, ***p < 0.001 between the indicated groups. Neutral. Abs means neutralizing antibodies. IgG was used as control (-) for neutralizing antibodies. **(D)** Schematic illustration of the major findings of this study.

Next, we determined the effect of BMM-CM on AMPK activation, SREBP-1c and SCD-1 expression in AML12 hepatocytes. In line with their effects on triglyceride synthesis, AMPK activation as monitored by phospho-AMPK-T172 in the hepatocytes was significantly higher when the cells were treated with Arg-II^−/−^-BMM-CM as compared to the cells treated with control WT-BMM-CM (Fig. 8A). BMM-CM pretreated with neutralizing antibodies against IL-6 and TNF-α elicited enhanced AMPK activation (Fig. 8A). In accordance, the expression levels of SREBP-1c and SCD-1 in the hepatocytes were significantly lower when the cells were treated with Arg-II^−/−^-BMM-CM as compared to the cells treated with control WT-BMM-CM (Fig. 8B,C). Pretreatment of BMM-CM with neutralizing antibodies against both cytokines further reduced the expression levels of SREBP-1c and SCD-1 (Fig. 8B,C).

## Discussion

In the present study, we provide both *in vitro* and *in vivo* evidences that Arg-II plays a role in HFD-induced liver steatosis through promoting macrophage inflammation in liver. Here we show that Arg-II^−/−^ mice exhibit significantly less hepatic steatosis with decreased triglyceride content, lower hepatic weight, reduced inflammatory cytokines and macrophage infiltration, which is accompanied by enhanced AMPK signaling and reduced expression/activity of hepatic lipogenic regulator and enzymes, including SREBP-1c, G6PDH, FAS and SCD-1, leading to mitigated *de novo* lipogenesis in the liver *in vivo*. The reduced liver steatosis in Arg-II^−/−^ mice on HFD is in line with the results of our previous study showing an improved glucose tolerance and insulin sensitivity in these mice^26^. The decreased hepatic steatosis in the Arg-II^−/−^ mice does not seem to be the result of decreased lipid delivery to the liver or increased lipid secretion from liver into the blood stream in form of lipoprotein, since there is no difference in plasma triglyceride levels between WT and Arg-II^−/−^ mice fed HFD as shown in our previous study^26^. Our data suggest that the reduced hepatic *de novo* lipogenesis in the obese Arg-II^−/−^ mice ends up in decreased intrahepatic triglyceride accumulation versus lipid secretion. A dissociation of plasma triglyceride levels from liver steatosis has been reported by numerous studies^36^,^37^. Moreover, we show that *in vitro*, the CM from Arg-II^−/−^ macrophages elicited lower triglyceride synthesis in cultured hepatocytes in the presence of oleic acid, which is accompanied by enhanced AMPK signaling and suppressed lipogenic regulator/enzyme SREBP-1c and SCD-1 and is attributable to the reduced production of the pro-inflammatory cytokines TNF-α and IL-6 from Arg-II^−/−^ macrophages. Our findings thus suggest that targeting Arg-II is beneficial for obesity-associated NAFLD.

In agreement with the previous studies on Arg-I and -II expression in various tissues^22^,^38^, we could not detect Arg-II expression in liver from mice by immunoblotting analysis, nor Arg-II was detectable in hepatocytes by immunostaining. Given that Arg-II-deficiency protects mice from obesity-associated fatty liver despite undetectable Arg-II in hepatocytes, we postulate that the protective effects are secondary to an effect on other tissues or cells. Studies have provided convincing evidence showing that liver macrophages including resident Kupffer cells and recruited myeloid cells from circulating blood, when activated, promote hepatic inflammation and accelerate hepatic lipogenesis leading to lipid accumulation in the hepatocytes through release of pro-inflammatory cytokines^17^,^19^. A recent study by Morinaga *et al*. elegantly demonstrated that, in HFD-induced obesity mouse model, Kupffer cells become activated to express MCP-1 (although the number of Kupffer cells does not increase), which then recruit macrophages from blood to liver, enhancing hepatic inflammation and hepatic insulin resistance^20^. In line with this study, we observed increased macrophages in the liver of obese WT mice, which was reduced in obese Arg-II^−/−^ mice. Importantly, Arg-II is mainly expressed and markedly enhanced in hepatic macrophages with concomitant enhanced accumulation of macrophages in obese WT mice as demonstrated by immunostaining, since the immunostaining signals of Arg-II in macrophages as well as the number of CLEC4F/Arg-II double positive or F4/80/Arg-II double positive cells are increased in the obese WT mouse liver. These observations are in accordance with our previous study^26^. Consistent with these observations, we also found significantly reduced TNF-α levels as well as reduced IL-6, MCP-1 and CCR-2 levels (although statistically not significant) in the liver of obese Arg-II^−/−^ mice as compared to the obese WT control animals. These findings demonstrate that Arg-II is associated with macrophage-mediated pro-inflammatory responses, which is in agreement with our previous findings that Arg-II is upregulated in macrophages of mice fed HFD and plays a causative role in promoting macrophage pro-inflammatory responses in obesity and insulin resistance^26^. Moreover, vascular inflammation and endothelial dysfunction associated with aging are protected in the Arg-II^−/−^ mice^27^. In support of our notions, the pro-inflammatory effects of Arg-II in endotoxin-induced uveitis and in diabetic nephropathy have been reported^39^,^40^. All the findings demonstrate a pro-inflammatory role of Arg-II in several inflammation disease models. In the present study, we further demonstrate that the Arg-II^−/−^-BMM-CM elicits a reduced triglyceride synthesis in the presence of oleic acid in cultured hepatocytes as compared to that of WT-BMM-CM, which is attributable to mitigated production of TNF-α and IL-6 from Arg-II^−/−^-BMM, since neutralizing antibody against TNF-α or IL-6 and combination of the two antibodies exhibit inhibition of triglyceride accumulation in the hepatocytes. These *in vitro* studies using AML12 hepatocytes were aimed to reinforce our findings *in vivo*. Although it is not optimal to use this cell line, which is established from a mouse hepatocyte transgenic for human TGF-α, the results on triglyceride accumulation and intracellular signaling in response to treatment with conditioned medium from WT and Arg-II^−/−^-BMM are in line with the *in vivo* experiments and support our conclusion. The fact that silencing Arg-II in monocytes decreased their adhesion activity to endothelial cells^26^ suggest that this may at least in part accounts for suppressed macrophage-infiltration in liver of Arg-II^−/−^ mice fed HFD. It is of great interest to further evaluate the precise mechanisms of Arg-II in regulating adhesion of monocytes to endothelial cells. Regarding the molecular mechanism regulating Arg-II expression in macrophages in obesity, evidence has been presented that hyperactive S6K1 upregulates Arg-II in cardiovascular system^27^,^28^. Taking into account that HFD has been reported to activate S6K1 in various tissues^41^,^42^, it is attempting to speculate that S6K1 may also mediate HFD-induced increase in Arg-II expression in macrophage. Further experiments will be required to verify this hypothesis. Taken together, our *in vivo* and *in vitro* experiments demonstrate that Arg-II deficiency protects mice from obesity-linked liver steatosis through suppression of macrophage-mediated hepatic inflammation.

Both mTORC1-S6K1 and AMPK pathways have been implicated in insulin resistance and lipogenesis in the liver in obesity at least in part through regulation of SREBP-1c gene expression and activation^4^,^5^,^6^,^7^,^43^. In the vascular cells, we showed a positive crosstalk between Arg-II and mTORC1-S6K1 as well as a negative crosstalk between Arg-II and AMPK pathway^27^,^28^,^29^. In the present study, we observed a higher hepatic AMPK signaling in obese Arg-II^−/−^ mice as compared to the obese WT mice, whereas no difference in hepatic mTORC1-S6K1 signaling between the two genotypes of obese mice was detected. Taking into account that AMPK inhibits mTORC1-S6K1 signaling pathway^44^, the fact that elevated AMPK signaling in Arg-II^−/−^ liver is not accompanied by reduced mTORC1-S6K1 signaling suggests that an AMPK-independent mechanism in activating mTORC1-S6K1 is dominant. Moreover, these results also suggest that elevated AMPK suppresses hepatic lipogenesis and ultimately liver steatosis through mTORC1-S6K1-independent mechanism(s) in obese Arg-II^−/−^ mice. Indeed, AMPK has been shown to suppress SREBP-1c expression and activity by directly phosphorylating SREBP-1c-S372^6^,^7^,^45^. Since Arg-II is not detectable in the liver of WT mice fed either NC or HFD, the difference in hepatic AMPK signaling between WT and Arg-II^−/−^ does not result from the crosstalk between Arg-II and AMPK as observed in vascular cells^29^. Adiponectin, an important adipocyte-derived factor that has inhibitory effects on insulin resistance, hepatic steatosis and inflammation^46^, is a well-known endogenous AMPK activator^47^. However, a role of adiponectin in activation of AMPK in the liver of obese Arg-II^−/−^ mice can be excluded, since no differences in circulating or hepatic levels of adiponectin were evident between obese WT and Arg-II^−/−^ mice, although adiponectin level was significantly higher in adipose tissue of Arg-II^−/−^ mice than the WT controls. Since there is no difference in epididymal fat weight between the obese Arg-II^−/−^ and WT mice, this suggests that the discrepancy of the difference in adiponectin levels in adipose tissue and plasma between the two genotypes is not a consequence of a decrease in fat mass in Arg-II^−/−^ mice. It rather indicates an increased local paracrine/autocrine but not endocrine secretion of adiponectin from adipose tissue into the circulation in Arg-II^−/−^ mice. The fact that suppressed macrophage-mediated hepatic inflammation accounts for the reduced lipogenesis and liver steatosis in Arg-II^−/−^ mice prompted us to hypothesize that the enhanced AMPK in hepatocytes is attributable to the reduced hepatic inflammation. The hypothesis is confirmed by our *in vitro* experiments showing that hepatocytes treated with the Arg-II^−/−^-BMM-CM exhibits higher AMPK activation, lower mRNA levels of SREBP-1c and SCD-1 as compared to the cells treated WT-BMM-CM, which could be further improved by neutralizing antibodies against IL-6 and TNF-α.

There is an increase in serum liver enzymes ALT and AST in Arg-II^−/−^ mice fed HFD, but this increase is actually smaller than those from WT-HFD group, although it does not reach statistical significance. Together with the results of hepatic steatosis and inflammation, these results demonstrate that Arg-II deficiency reduces but does not abolish liver injury in HFD-induced obesity. Extensive studies have shown that inhibition of SCD-1 in the context of the overflow of free fatty acids coming to the liver on HFD feeding causes the build-up of toxic lipids resulting in increased hepatocyte cell death and liver injury even there is decreased triglyceride deposition^32^,^33^. However, we show here that significantly reduced SCD-1 in the liver of obese Arg-II^−/−^ mice as compared to that of obese WT mice does not lead to an accelerated liver injury while suppressing hepatic triglyceride synthesis, suggesting that the decreased hepatic SCD-1 is not necessarily accompanied by an exacerbation of liver damage. It remains elusive what is the mechanism that prevents hepatocyte damage from a decreased hepatic SCD-1 level under certain conditions such as in Arg-II^−/−^ mice.

During preparation of this manuscript, two studies on the role of arginase in obesity-associated liver steatosis with opposite conclusions were reported^30^,^31^. In the study of Moon *et al*.^30^, using arginase inhibitor nor-NOHA that is an isoform non-specific inhibitor, the authors showed some similar findings to the ones we obtained with Arg-II^−/−^ mice including reduced liver steatosis and liver triglyceride content, reduced expression of SCD-1 as well as elevated AMPK signaling. By demonstrating the direct inhibitory effect of nor-NOHA on oleic acid-induced triglyceride synthesis with concurrent increase in NO production and AMPK signaling in HepG2 hepatocytes *in vitro*, they conclude that arginase inhibition reduces obesity-induced hepatic lipid abnormality most likely attributable to the inhibition of Arg-I. However, this mechanism unlikely accounts for the beneficial effect of Arg-II deficiency in obesity-induced liver steatosis in our study, since Arg-II is not expressed in hepatocytes and no change in Arg-I level or total arginase activity in liver of Arg-II^−/−^ mice was observed. In contrast to this study and also to our present study, Navarro *et al*. using Arg-II^−/−^ mice, reported an increased macrophage inflammation and liver steatosis accompanied by an increase in *de novo* hepatic lipogenesis in Arg-II^−/−^ mice on HFD feeding. Although reasons of the discrepancy between the studies are not clear, yet, the following points could be taken into consideration. In the study by Navarro *et al*. all the control C57BL/6J mice were purchased from Jackson laboratory, whereas the Arg-II^−/−^ mice were obtained from another laboratory. In our study, we used offspring of WT/WT and Arg-II^−/−^/Arg-II^−/−^ derived from heterozygous breeding. Evidence has been presented that multiple substrains will be created within an inbred strain that became increasingly more genetically divergent through generations of independent inbreeding^48^. Although Arg-II^−/−^ mice have been backcrossed to C57BL/6J mice over 10 generations, which is considered as congenic to C57BL/6J, the independent breeding of the mice may lead to creation of a substrain that became genetically divergent from C57BL/6J mice purchased from Jackson Laboratory. It could thus not be excluded that the difference in macrophage inflammation associated with liver steatosis phenotype observed in their study is consequence of other genetic differences. Moreover, the numbers of animals used were not indicated throughout their report. If the animal number used in the study is too small, the results could be biased. In addition, Arg-II is highly expressed in the liver of WT type mice in their report, which is in contrast to studies published by other groups including our present study^22^,^38^. It is puzzling that Arg-II is even detectable in the Arg-II^−/−^ liver, although it is at lower levels as compared to the WT mice. This raises concerns about the genetic modified mouse model used in their study. Nevertheless, despite very few reported studies on the role of Arg-II in macrophage inflammatory responses, our work showing the pro-inflammatory role of Arg-II in macrophages is in accordance with several other studies^25^,^39^,^40^. Finally, our previous studies also provide convincing evidence showing a protective and healthier metabolic and aging phenotype of Arg-II^−/−^ mice on HFD or high cholesterol diet^26^,^27^,^28^,^29^.

In summary, our present study demonstrates that targeting Arg-II protects mice from HFD-induced liver steatosis through suppression of macrophage inflammation and suppression of the release of TNF-α and IL-6, which leads to improved AMPK activation, resulting in inhibition of SREBP-1c and ultimately the suppression of lipogenic enzymes and inhibition of hepatic lipogenesis (Fig. 8D). Our findings implicate that targeting Arg-II is beneficial for obesity-associated NAFLD.

## Methods

### Materials

All chemicals including those used for immunoblotting and anti-tubulin (T5168) were obtained from Sigma (Buchs, Switzerland); anti-phospho-AMPK (Thr-172) (2535S), anti-AMPKα (2793S), and anti-SCD-1 (2438S) were from Cell Signaling (Allschwil, Switzerland); anti-Arg-II (sc-20151) and anti-CLEC4F (SC-242467) were from Santa Cruz (Nunningen, Switzerland). Anti-F4/80 (MCA497G) was from AbD Serotec (Dusseldorf, Germany). Bio-Rad DC^TM^ protein assay kit was from Bio-Rad (Basel, Switzerland); Alexa Fluor680-conjugated anti-mouse IgG (A21057) was from Molecular Probes ⁄ Invitrogen (Lucerne, Switzerland); IRDye800-conjugated anti-rabbit IgG (926-32211) were from LI-COR Biosciences (Bad Homburg, Germany). Neutralizing rat anti-mouse-IL-6 (BMS178) and control rat IgG1 (16-4301-85) were from eBioscience (Vienna, Austria). Neutralizing anti-TNF-α (AB-410-NA) was from R&D systems (Minneapolis, USA). All reagents for the activity assay of lipogenic enzymes including ME, G6PDH and FAS were kindly provided by Jean-Francois Cajot (University of Fribourg, Switzerland).

### Animals

The breeding and genotyping of wild type (WT) and Arg-II-deficient mice (Arg-II^−⁄−^) were as previously described^26^,^49^. Starting at the age of 7 weeks, the male WT and Arg-II^−/−^ mice were given free access for 14 weeks to a HFD (energy content: 55% fat, 21% protein, and 24% carbohydrate, Harlan Teklad TD 93075) and maintained on a 12-h light-dark cycle and tap water. After 14 weeks of HFD, the animals were sacrificed after intraperitoneal anesthesia with xylazine (10 mg kg^−1^body weight) and ketamine (100 mg kg^−1^ body weight). Blood and liver tissues were then collected for histological analysis or snap-frozen in liquid nitrogen and kept at −80 °C until processed. Animal work was approved by the Ethical Committee of Veterinary Office of Fribourg (number 174/07), Switzerland and was performed in compliance with guidelines on animal experimentation at our institution.

### Cell Culture

The mouse AML12 hepatocytes cell line from American Type Culture Collection were maintained in DMEM/F-12 Ham medium supplemented with 10% fetal bovine serum (FBS) and human insulin- transferrin- sodium selenite (ITS) media supplement. Treatments were carried out after 6 hours starvation in 0.2% bovine serum albumin (BSA)-ITS-DMEM-F-12 Ham.

Bone marrow-derived macrophages (BMM) were prepared from bone marrow-derived cells by incubation with 10% HIFBS-RPMI, supplemented with 20% of L929-conditioned medium containing macrophage colony–stimulating factor, for 6 to 7 days to induce differentiation as described previously^26^. After 6 hours starvation in 0.2% BSA–RPMI, cells were either untreated or treated with LPS (100 ng/ml, 8 h). The conditional medium (CM) was then collected for further study.

### Enzyme-Linked Immunosorbent Assay (ELISA)

The plasma adiponectin levels was evaluated according to the manufacturer’s instruction with the Adiponectin ELISA Kit (Assaypro LLC, Saint Charles, Missouri, USA), the IL-6 and TNF-α level in BMM-CM were with the ELISA MAX^TM^ Deluxe kit from BioLegend (Lucerna Chem AG, Luzern, Switzerland).

### Real-Time Quantitative Reverse Transcription Polymerase Chain Reaction Analysis (qRT-PCR)

Total RNA was extracted from tissues with Trizol Reagent (Molecular Research Center, Inc., Cincinnati, OH, USA) following the supplier’s protocol. The mRNA expression was evaluated by two-step qRT-PCR analysis as described previously^26^. The mRNA expression levels of all genes were normalized to the reference gene glyceraldehyde 3-phosphate dehydrogenase (GAPDH). The primer sequences are as follows:

mIL-6F: GACAACCACGGCCTTCCCTA ;

mIL-6R: GCCTCCGACTTGTGAAGTGGT.

mTNF-αF: GGCAGGTCTACTTTGGAGTCATTGC;

mTNF-αR: ACATTCGAGGCTCCAGTGAATTCGG.

mMCP-1F: AGCACCAGCCAACTCTCAC;

mMCP-1R: TCTGGACCCATTCCTTCTTG.

mAdiponectinF: TGTTCCTCTTAATCCTGCCCA;

mAdiponectinR: CCAACCTGCACAAGTTCCCTT.

mGAPDHF: ACCCAGAAGACTGTGGATGG;

mGAPDHR: ACACATTGGGGGTAGGAACA.

mSREBP-1cF: AAGCAAATCACTGAAGGACCTGG

mSREBP-1cR: AAAGACAAGGGGCTACTCTGGGAG

mSCD-1F: TTCTTACACGACCACCACCA

mSCD-1R: GCAGGAGGGAACCAGTATGA

### Interaction between BMM and Hepatocytes *in vitro*

After 6 h starvation, AML12 hepatocytes were changed to BMM-CM and incubated in presence of oleic acid (OA, 20 mmol/L) for 24 h till the extraction.

For antibody blocking studies, the BMM-CM was incubated with control IgG or neutralizing anti-IL-6 (18 μg/ml), anti-TNF-α (0.4 μg/ml) or combination of anti-IL-6 and anti-TNF-α for 2h prior to the addition to the AML12 cells as described above.

### Measurement of Triglyceride Levels in Cultured Hepatocytes and in Liver Tissues

The lipid extraction and triglyceride measurement in cultured hepatocytes were carried out with the triglyceride quantification kit (BioVision, Mountain View, CA) according to the manufacturer’s instructions. Measurement of liver triglyceride was performed as previously described^50^. Briefly, Livers were homogenized and the hepatic triglyceride was extracted by digesting 100–300 mg liver tissues overnight at 55 °C in 350 μl ethanolic potassium hydroxide (2:1 v/v of 100% ethanol and 30% potassium hydroxide). Samples were neutralized with water:ethanol (1:1 v/v; total volume of 1 ml). After centrifugation (8000 g, 5 min), 1 ml of supernatant was collected and brought to 1.2 ml volume with water:ethanol (1:1). Then, 200 μl was removed and proceeded to saponification with 215 μl of 1 mol/L MgCl_2_ by vortexing and incubating on ice for 10 min. Saponified liver extracts (the upper phase) were separated by centrifugation and kept frozen at −80 °C. After thawing the samples, triglyceride quantity was determined by an enzymatic colorimetric assay (TRIGL:ACN 781) on Roche/Hitachi cobas c system (Roche, Switzerland). The color intensity of the red dye stuff formed was proportional to the triglyceride concentration (mmol/L) and was determined at 505 nm.

### Lipogenic Enzyme Activity Assay

To assess the activities of the lipogenic enzymes including ME, G6PDH and FAS in liver, liver tissues were homogenized in extraction buffer consisting of 250 mmol/L sucrose, 50 mmol/L Tris/HCl pH 7.3 and 30 mmol/L mercaptoethanol, and centrifuged at 13000 rpm for 10 min at 4 °C. Protein concentrations of the supernatants were determined by Bradford assay solution from Applichem (A6932, 0500).

The activity of ME and G6PDH was determined spectrophotometrically by measuring the increase in absorbance at 340 nm resulted from the reduction of NADP as previously described^51^,^52^, whereas FAS activity was determined spectrophotometrically by measuring the decrease in absorbance at 340 nm resulted from the oxidation of NADPH as described previously^53^. All the enzyme activity assays were run at 37 °C in duplicate in a prewarmed Bio-Rad (Model 680) microplate Spectrophotometer. Absorbance at 340 nm (37 °C) was measured using software programmed for a kinetic read sequence of 1 min intervals over 10 minutes for ME and G6PDH, every 2 min over 20 minutes for FAS. The enzyme activity was calculated by obtaining the ΔA340 nm/minute with the maximum and the minimum value using 5 data points in the linear range with respect to time and to sample concentration. Data are presented as fold change in the enzyme activity.

### Immunoblotting Analysis

Cell lysate preparation, SDS-PAGE, immunoblotting, antibody incubation and signal detection were performed as described previously^26^. Quantification of the signals was performed using NIH Image 1.62 software.

### Hematoxylin and eosin (HE) and Oil Red O Staining

Liver tissues embedded in paraffin or frozen in OCT compounds were cut at 7 μm, then stained with HE or oil red O, respectively, as described previously^26^,^54^.

### Tissue Immunofluorescence Staining and Confocal Microscopy

Preparation of paraffin-embedded sections (7 μm) and immunostaining were performed as described previously^26^. The immunofluorescence signals were visualized under LEICA’s DIM6000 Confocal microscope. The fluorescence signals were quantified with NIH IMAGE J and results are presented as the ratio of F4/80, Arg-II, CLEC4F to DAPI-positive nucleus.

### Measurement of blood parameters

Measurement of plasma alanine aminotransferase (ALT) and aspartate aminotransferase (AST) were performed by Laboratory HFR, Hospital Fribourgeois. Plasma adiponectin was determined by ELISA.

### Statistics

Data are given as mean±SEM. In all experiments, n represents the number of animals. Statistical analysis was performed with unpaired t test or ANOVA with Dunnett or Bonferroni post-test. Differences in mean values were considered significant at p ≤ 0.05.

## Additional Information

**How to cite this article**: Liu, C. *et al*. Targeting arginase-II protects mice from high-fat-diet-induced hepatic steatosis through suppression of macrophage inflammation. *Sci. Rep*. **6**, 20405; doi: 10.1038/srep20405 (2016).

## Supplementary Material

Supplementary Information
