# Supplementary material for: Targeting arginase-II protects mice from high-fat-diet-induced hepatic steatosis through suppression of macrophage inflammation
Source: Sci Rep. 2016 Feb 5;6:20405. doi: 10.1038/srep20405 (PMC4742779; doi:10.1038/srep20405)
Supplement: Supplementary Information [file srep20405-s1.pdf]

Scientific Reports
December 1st, 2015
SREP-15-20951B

Targeting arginase-II protects mice from high-fat-diet-induced hepatic steatosis through suppression of macrophage inflammation

Chang Liu[1], Angana G. Rajapakse[1], Erwin Riedo[2], Benoit Fellay[2], Marie-Claire Bernhard[3], Jean-Pierre Montani[1,4] , Zhihong Yang[1,4*] and Xiu-Fen Ming[1,4*]

[1]Vascular Biology, Department of Medicine, Division of Physiology, University of Fribourg, Chemin du Musée 5, CH-1700 Fribourg, Switzerland

[2] Laboratory HFR, Hospital Fribourgeois, Chemin des Pensionnats 2, 1708 Fribourg, Switzerland

[3] Promed Medical Laboratory SA, Fribourg, Switzerland

[4]National Center of Competence in Research "Kidney.CH", Switzerland

Address for correspondence:

Zhihong Yang, MD or
Xiu-Fen Ming, MD, PhD
Laboratory of Vascular Biology
Department of Medicine
Division of Physiology
University of Fribourg
Chemin du Musée 5
CH-1700, Fribourg
Switzerland
Tel: 0041-26-300 85 93
Fax: 0041-26-300 97 34
Email: zhihong.yang@unifr.ch
or xiu-fen.ming@unifr.ch

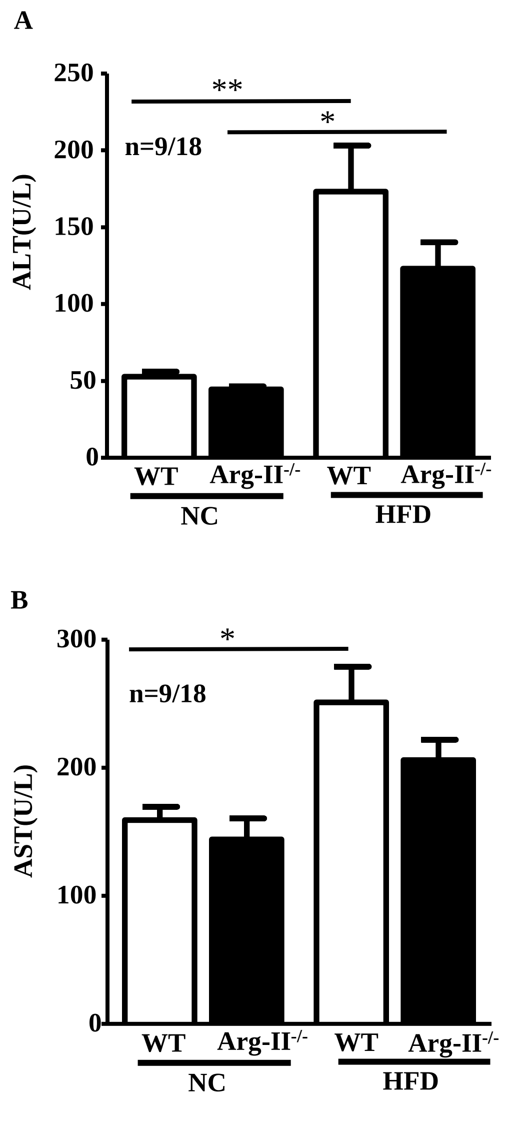

Fig. S1. The increase in plasma ALT and AST tends to be smaller in obese Arg-II$^{-/-}$ mice as compared to obese WT mice, but does not reach statistical significance. Liver injury was assessed by measuring plasma ALT **(A)** and AST **(B)**. n=9 (NC) and n=18 (HFD).

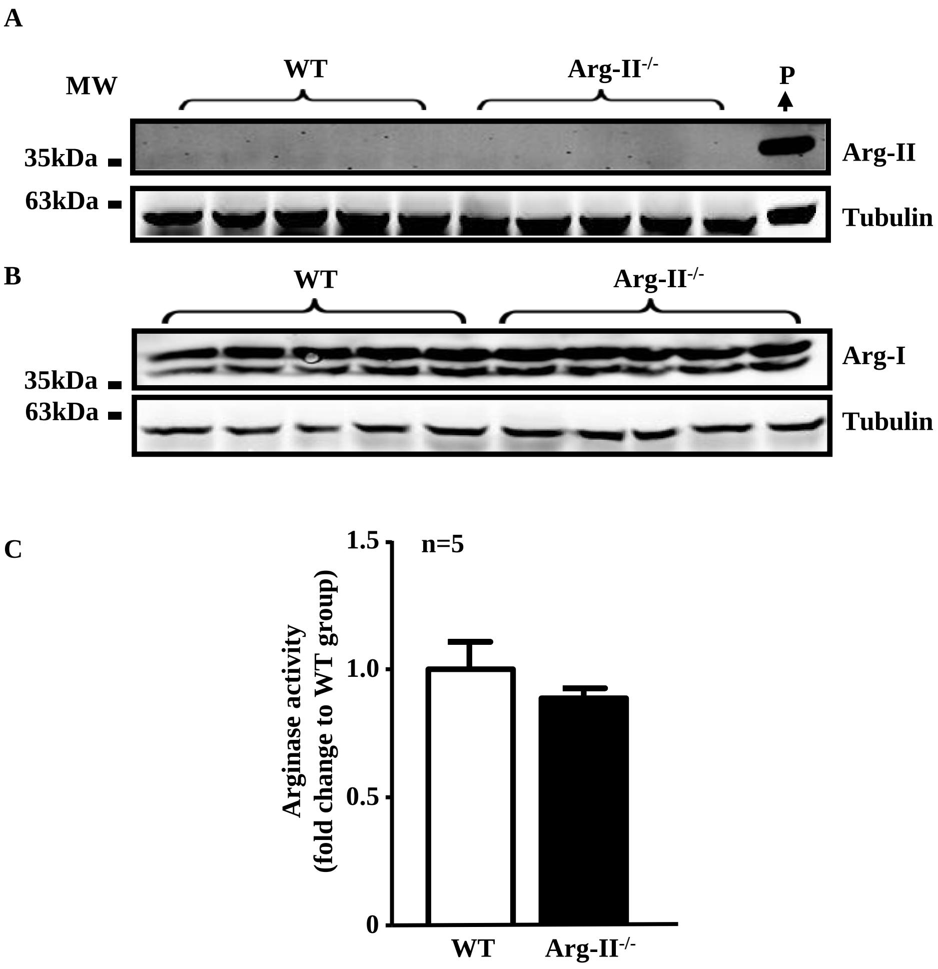

Fig. S2. Arg-II is not detectable in liver lysates, whereas Arg-I expression and total arginase activity in liver of obese mice are not significantly affected by Arg-II-deficiency. Liver lysates from obese WT and Arg-II$^{-/-}$ mice were prepared and subjected to immunoblotting analysis of (**A**) Arg-II expression. Mice kidney lysate was used as positive control (P). **(B)** Arg-I expression. Tubulin served as loading control. (**C**) Arginase activity assay.

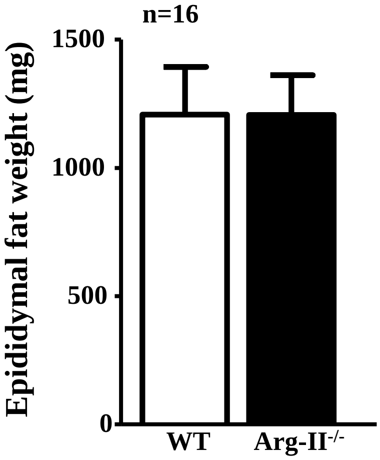

Fig. S3. There is no significant difference in epididymal fat weight between wt and Arg-II$^{-/-}$ mice fed HFD.

A.

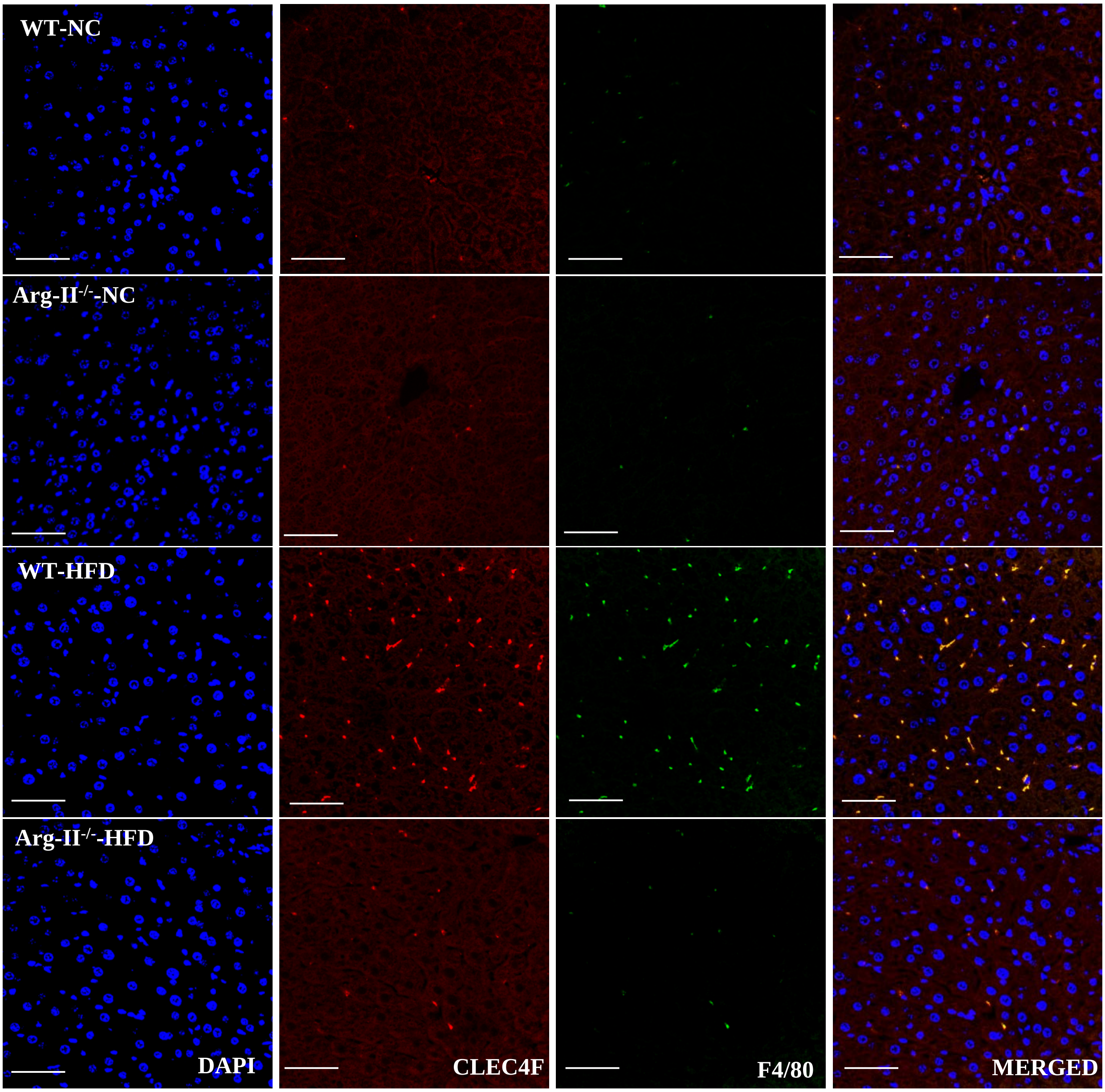

B.

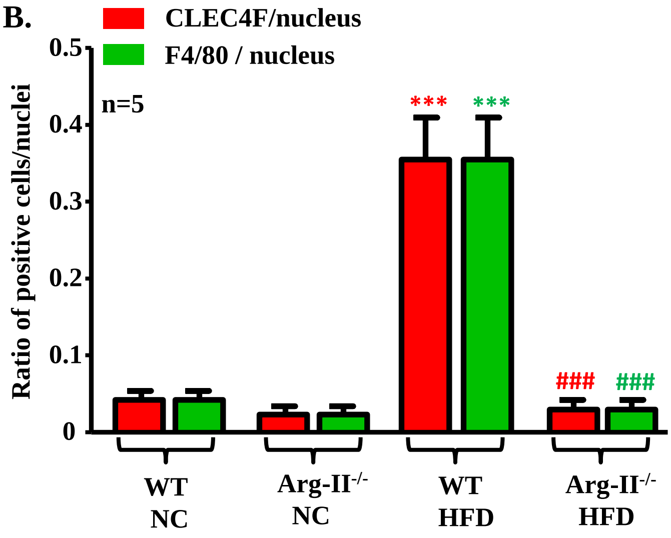

Fig. S4. All the macrophages in liver are Kupffer cells. (A) Liver sections from WT and Arg-II$^{-/-}$ mice fed NC or HFD were stained with pan-macrophage marker F4/80 (green) and Kupffer cell specific marker CLEC4F (red) followed by nuclei counterstaining with DAPI (blue). **(B)** Quantification of the fluorescence signals.
